# Astrocytes from P301S Tau mice exhibit non-canonical protein secretion and reduced morphological complexity

**DOI:** 10.4103/NRR.NRR-D-24-01598

**Published:** 2025-08-13

**Authors:** Aishwarya G. Nadadhur, Matthew Mason, Johanna S. Rees, Marta Sidoryk-Wegrzynowicz, Aviva M. Tolkovsky, Maria Grazia Spillantini

**Affiliations:** 1 Department of Clinical Neurosciences, University of Cambridge, Cambridge, UK; 2 Department of Biochemistry, University of Cambridge, Cambridge, UK; 3 Laboratory of Pathoneurochemistry, Department of Neurochemistry, Mossakowski Medical Research Center, Polish Academy of Sciences, Warsaw, Poland

**Keywords:** astrocyte conditioned medium, basal metabolism, extracellular matrix, nerve regeneration, neuronal survival, P301S tau transgenic mice, structural maturation, tau, tauopathy, unconventional secretion

## Abstract

Astrocytes have important neurosupportive functions in the brain that are altered in neurodegenerative diseases by unresolved mechanisms. We showed previously that astrocytes cultured from mice transgenic for human P301S-tau (P301S-mice) recapitulate the deficit in production and secretion of thrombospondin1 found in symptomatic P301S mouse brains, causing both reduced synapse formation and survival of cultured neurons. To further characterize how P301S-derived astrocytes differ from controls, we have compared the astrocyte-conditioned media of cultured astrocytes from postnatal day 7/8 P301S mice (P301S-astrocyte-conditioned media) *versus* controls (C57-astrocyte-conditioned media) using label-free liquid chromatography-mass spectrometry. We verified that thrombospondin1 secretion was significantly reduced in the P301S-astrocyte-conditioned media *versus* C57-astrocyte-conditioned media, demonstrating the robustness of the analysis. The most notable distinction was that **~**57% of the P301S-astrocyte-conditioned media-enriched proteins were cytoplasmic proteins linked to cellular metabolism that are not predicted to be secreted via classical or non-classical secretion pathways, whereas **~**88% of C57-astrocyte-conditioned media-enriched proteins comprised classically secreted proteins enriched in extracellular matrix components. These differences are associated with the finding that P301S-derived cultured astrocytes were smaller and *in vivo* appeared less mature in the cortex of P301S mice. The unconventional secretion pathway that P301S-astrocyte-conditioned media display shares similarities with several amyloid-β-exposed astrocyte-conditioned media, indicating that stimuli induced by tau and amyloid-β may induce a common adverse response pathway. Altogether, members of this adverse pathway may serve as a potential set of biomarkers to aid the clinical diagnosis of Alzheimer’s disease and other tauopathies, while the list of reduced neurosupportive factors could indicate new approaches to enhance neuronal survival by factor supplementation in tauopathies.

## Graphical abstract

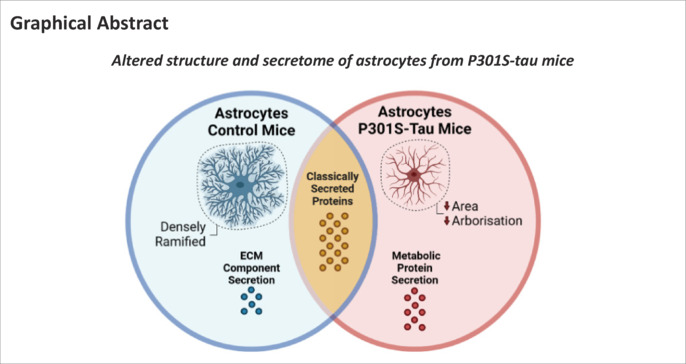

## Introduction

The tauopathies comprise a large group of human neurodegenerative diseases that are characterized by the aggregation of the microtubule-associated protein tau into post-translationally modified, hyperphosphorylated, insoluble filamentous inclusions (Spillantini and Goedert, 2013; Goedert and Spillantini, 2019). Tau fibrils acquire disease-specific conformations (Shi et al., 2021) that may spread in a prion-like manner through the nervous system as disease progresses (Goedert, 2020). The identification of disease-causing mutations in *MAPT*, the tau gene, in frontotemporal dementia and Parkinsonism-linked to chromosome 17 has established that dysfunction of tau is central to the neurodegenerative process (Ghetti et al., 2015; Goedert et al., 2024). Indeed, tau neurofibrillary tangles are also best correlated with neuronal cell death and dementia in sporadic Alzheimer’s disease (Braak and Braak, 1991; Arriagada et al., 1992; Tanner et al., 2022) and other sporadic dementias (Ruchika et al., 2023).

Although misfolded hyperphosphorylated tau is critical for the development of disease and neuronal death, the mechanisms of tau-related toxicity are still not clear. P301S tau transgenic mice (P301S mice) that express mutant human 0N4R tau specifically in neurons under the control of the neuronal Thy1.2 promoter were developed to study the mechanisms of tau toxicity (Allen et al., 2002). These mice develop early behavioral signs of pathology within a week after birth (Scattoni et al., 2010) after which disease severity increases until neuronal tau aggregates, which are associated with neuronal death (Delobel et al., 2008; Macdonald et al., 2019), are found in many brain regions including the superficial layers of the motor cortex (Hampton et al., 2010), the perirhinal and piriform cortices (Romberg et al., 2013; Yang et al., 2015) and the spinal cord and peripheral nervous system (Allen et al., 2002; Mellone et al., 2013). This process of aggregation begins as early as 5 weeks of age, with the emergence of neurofibrillary tangles by 2–3 months of age, and a progressive increase in neurofibrillary tangle burden over time. Recent data have implicated non-autonomous roles of astrocytes and microglia in enhancing the pathology of tau (Hartnell et al., 2021). The terms ‘activation’ and ‘reactive gliosis’ are used to describe states of glial cells that are associated with disease progression in almost all human neurodegenerative diseases (Mohn and Koob, 2015; Pekny et al., 2016; Amlerova et al., 2024). In particular, astrogliosis appears to precede neuronal loss in tauopathies, suggesting an important contribution of astrocytes to disease development (Leyns and Holtzman, 2017; Sidoryk-Wegrzynowicz et al., 2017; Sidoryk-Wegrzynowicz and Struzynska, 2019). In P301S mice, a deficiency of survival support by the endogenous astrocytes, coupled with a possible gain of toxic functions (Guttenplan et al., 2021), was indicated by rescue of neurons in the superficial layers of the motor cortex after transplanting neuron precursor cell–derived astrocytes (Hampton et al., 2010). A similar deficit intrinsic to the astrocytes was indicated by decreased survival and reduced synapse abundance of cultured neurons when exposed to the conditioned medium from P301S-derived astrocytes (henceforth P301S astrocytes) compared to controls (henceforth C57 astrocytes) (Sidoryk-Wegrzynowicz et al., 2017). A reduction of thrombospondin1 (Thbs1) was found in the cortex of P301S mice with neurofibrillary tangles and this was paralleled by its reduced abundance in the secretome of cultured astrocytes from P301S mouse brain compared to controls. This reduction in Thbs1 contributed to neuronal deficit *in vitro* as demonstrated by the substantial recovery of neuronal growth and survival upon the addition of exogenous Thbs1 (Sidoryk-Wegrzynowicz et al., 2017).

In addition to finding Thbs1 deficits in the secretome, we hypothesized that the secretory profiles of astrocytes derived from neonatal P301S mice may show other compositional changes related to the presence of human mutant tau. Here, we have aimed to characterize the differences between the secretory profiles of astrocytes derived from neonatal P301S mice and age-matched controls, and the implications these differences may have on astrocyte and neuron function.

## Methods

### Astrocyte culture

Astrocytes were prepared from homozygous 7–8-day-old C57BL/6JOlaHSD or P301S-OlaHSD mice on the OlaHSD background (C57Bl-6jOlaHSD strain, Inotiv, West Lafayette, IN, USA), P301S-tau strain Tg(Thy1-MAPT*P301S)2541Godt) (Allen et al., 2002; Mellone et al., 2013) of both sexes as described (Sidoryk-Wegrzynowicz et al., 2017) with minor differences. Briefly, after decapitation, brains were collected into ice-cold HBSS, meninges were removed, and cortices were isolated. Cortical tissue was cut into **~**1 mm pieces and digested for 20 minutes in 1 mL in a solution containing 0.25% Trpysin/EDTA (Cat# 25200072, Gibco, Altrincham, Cheshire, UK) buffered with 0.35 g/L bicarbonate in a 5% CO_2_ incubator. Digestion medium was replaced with astrocyte culture medium comprising DMEM/F12, 10% heat-inactivated FBS (Cat# 10082147, Gibco), Glutamax (Cat# 35050061, Gibco), antibiotic-antimycotic (Cat# 15240062, Gibco), supplemented with 20 µg/mL DNase I (Cat# 1128493200, Sigma, Gillingham, Dorset, UK), triturated 10–15 times using a 1 mL Gilson pipette tip and filtered through a 70 µm mesh Cell Strainer (Cat# 08-771-2, Thermo Fisher Scientific, Altrincham, Cheshire, UK). After spinning at 300 × *g* for 3 minutes, the supernatant was aspirated, and the pellet was resuspended in an astrocyte culture medium. Cells were plated in T25 flasks coated with poly-L-lysine (10 µg/mL in water overnight, followed by 3 washes with water) for 7–10 days after which the cells were transferred to poly-L-lysine-coated T75 flasks. After 10–14 days, 5 µM PLX3397 (colony stimulating factor 1 receptor inhibitor Pexidartinib; Cat# S7818, Selleckchem, Waltham Abbey, Essex, UK) was added to the medium to remove microglia. Medium containing PLX3397 was changed twice weekly, and astrocyte conditioned medium (ACM) was harvested when cultures became confluent (after 30–40 days) and microglia had been cleared. Before ACM collection, cells were imaged using a 20× objective on an inverted Leica microscope. This study was conducted under Animal Project Licence number PP4466737, granted February 2, 2021. Animal experiments complied with the U.K. Animals (Scientific Procedures) Act, 1986 and associated guidelines (EU Directive 2010/63/EU for animal experiments) following the recommendations listed in The PREPARE Guidelines (Planning Research and Experimental Procedures on Animals: Recommendations for Excellence) for planning animal experiments (https://norecopa.no/prepare).

### Astrocyte-conditioned media preparation

Confluent cultures of astrocytes were washed thoroughly four times with preheated PBS containing Ca^2+^ and Mg^2+^ (DPBS; Cat# 1404011, ThermoFisher) to remove all traces of serum, and incubated in 6 mL astrocyte culture medium containing 15 mM Hepes without serum or phenol red. After 4 days, ACM was collected, filtered through a Millex 0.2 µm filter (#SLFG025LS, Millipore, Fairham, Nottingham, UK) and spun at 300 × *g* for 8 minutes. The supernatant was collected and supplemented with 1× Halt protease inhibitor (Cat# 78440, Thermo Fisher Scientific), and either concentrated immediately or snap frozen. Cleared ACM was concentrated through a 4 mL capacity Amicon® Ultra-4 Centrifugal Filter (Cat# UFC801024, Millipore) with a 10 KDa cutoff, by centrifugation at 4000 × *g* in a swinging bucket rotor (Cat# 75004230, Heraeus Labofuge 400R, Thermo Fisher Scientific) until the ACM was concentrated to 1.5–2 µg/µL.

### Proteomic sample preparation

Concentrated ACM was heated at 95˚C for 5 minutes in Laemmli sample buffer containing β-mercaptoethanol and 48 µL was run for 1 cm into a 10% Mini-PROTEAN TGX gel (Cat# 4561034, BioRad, Fisher Scientific, Loughborough, Leicestershire, UK). The gel was stained with InstantBlue (Cat# ISB1L, Sigma). Gel images can be found in **Additional Figure 1**. Each gel lane was cut into two bands, each of which was cut into 1 mm^2^ pieces, destained in water, reduced with 100 mM DTT (Cat# D0632, Sigma), alkylated with 100 mM iodoacetamide (Cat# I1149, Sigma) and digested overnight at 37°C with sequencing grade 0.5% Trypsin in ammonium bicarbonate (Cat# V5111, Promega, Southampton, Hampshire, UK). After digestion, the supernatant was brought to 0.1% formic acid and loaded onto an autosampler (Thermo Fisher Scientific) for automated liquid chromatography-mass spectrometry (LC-MS/MS) analysis.

**Figure 1:**
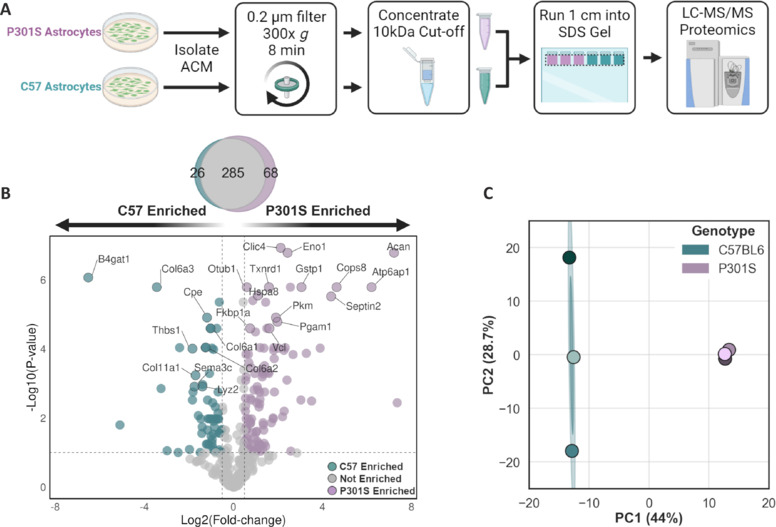
Differential enrichment of astrocyte-conditioned media (ACM) proteins secreted by P301S and control astrocytes. (A) Scheme of ACM preparation method. (B) Venn diagram and Volcano plot of the Log2(fold-change) of protein abundance between genotypes, highlighting proteins that were significantly enriched (false discovery rate < 0.1) in either P301S (pink) or C57 (teal) (*n* = 3 of each genotype). (C) Principal component analysis plot of all proteins represented in B, showing robust separation of the two genotypes across the PC1 axis. LC-MS/MS: Liquid chromatography-mass spectrometry; SDS: sodium dodecyl sulfate.

### Liquid chromatography-mass spectrometry analysis

All LC-MS/MS experiments were performed using a Dionex Ultimate 3000 RSLC nanoUPLC (Cat# 6041.7903K, Thermo Fisher Scientific) system and a Q Exactive Orbitrap mass spectrometer (Cat# 0726090, Thermo Fisher Scientific). Separation of peptides was performed by reverse-phase chromatography at a flow rate of 300 nL/min and a ThermoFisher reverse-phase nano Easy-spray column (Thermo PepMap C18, 2 µm particle size, 10 nm pore size, 75 µm internal diameter× 50 cm length). Peptides were loaded onto a pre-column (ThermoFisher PepMap 100 C18, 2 µm particle size, 10 nm pore size, 300 µm i.d. × 5 mm length) from the UltiMate 3000 autosampler (Cat# WPS-3000TPLRS, Thermo Fisher Scientific) with 0.1% formic acid for 3 minutes at a flow rate of 10 µL/min. After this period, the column valve was switched to allow the elution of peptides from the pre-column onto the analytical column. Solvent A consisted of 0.1% formic acid in water, and solvent B of 80% acetonitrile, 20% water with 0.1% formic acid. The linear gradient used was 2%–40% B in 90 minutes (the total run time including column washing and re-equilibration was 120 minutes). The LC eluant was sprayed into the mass spectrometer by means of an Easy-spray source (Thermo Fisher Scientific). All m/z values of eluting ions were measured in an Orbitrap mass analyzer, set at a resolution of 70,000 and scanned between m/z 380–1500. Data dependent scans (Top 20) were used to automatically isolate and generate fragment ions by higher energy collisional dissociation (HCD; normalized collision energy (NCE):32.5%) in the HCD collision cell and measurement of the resulting fragment ions was performed in the Orbitrap analyzer, set at a resolution of 35,000. Singly charged ions and ions with unassigned charge states were excluded from being selected for MS/MS and a dynamic exclusion of 60 seconds was employed.

### Database searching

Post-run, all MS/MS data were converted to Mascot Generic Fomrat files and the files were then submitted to the Mascot search algorithm (MatrixScience, version 2.6.0; https://www.matrixscience.com) and searched against the UniProt mouse database (61295 sequences; 27622875 residues; https://www.uniprot.org) and a common contaminant sequences containing non-specific proteins such as keratins and trypsin (123 sequences; 40594 residues). Variable modifications of oxidation (M) and deamidation (NQ) were applied as well as a fixed modification of carbamidomethyl (C). The peptide and fragment mass tolerances were set to 20 ppm and 0.1 Da, respectively. A significance threshold value of *P* < 0.05 and a peptide cut-off score of 20 were also applied. All data (DAT files) were then imported into the Scaffold program (Version_4.5.4, Proteome Software; https://www.proteomesoftware.com). Label-free quantification was performed, using the Scaffold emPAI algorithm.

### Data analysis

All data analysis was performed in Python (v. 3.8.12; https://www.python.org/). In total, 465 proteins were identified for which more than 2 unique peptides were detected by MS. Of these proteins, only 379 were retained after omitting proteins that were absent in more than 1 of the 3 samples per genotype. Missing value imputation was performed for proteins that were absent in 1 of 3 samples, using the mean of the other 2 samples. Data were normalized by genotype-specific quantile normalization (Zhao et al., 2020).

Pathway enrichment analysis was performed by submitting proteins to String-DB (https://string-db.org/), using an interaction confidence of 0.7, and exporting the Gene Ontology Biological Process (GOBP), Cellular Compartment (GOCC), and (Molecular Function) GOMF data. To predict protein signal peptides and protein localization, Fasta format primary sequences were retrieved programmatically using the UniProt API in Python. Fasta sequences were submitted to SecretomeP (https://services.healthtech.dtu.dk/services/SecretomeP-2.0/) and DeepLoc (https://services.healthtech.dtu.dk/services/DeepLoc-2.0/), for signal peptide prediction and protein localization predictions, respectively. Figures were generated with the Matplotlib (v. 3.7.1; https://matplotlib.org) and Seaborn (v. 0.13.0; https://seaborn.pydata.org) Python libraries, with the exception of the Volcano Plot, which was generated in R (v. 4.3.2; https://www.r-project.org) using the EnhancedVolcano library (v. 1.20.0; https://bioconductor.org).

### Cell area and morphology analysis

Images of astrocyte cultures were captured at the point of ACM collection and analyzed using ImageJ (Fiji Version 2.14.0/1.54f) by tracing the perimeters of over 240 cells per culture per genotype from 2 independent cultures to investigate whether there may be some structural abnormalities that underpin the differences in the secretome. Measurements were normalized by z-score and statistical significance between the two genotypes was tested on the cumulative probability distribution using a two-sample Kolmogorov-Smirnov test with the Scipy library (v. 1.10.1; https://scipy.org/) in Python. To compute the size and complexity of astrocytes *in vivo*, the Sholl plugin in ImageJ was used (https://imagej.net/imagej-wiki-static/Sholl) (Ferreira et al., 2014). Brain slices (25 µm) from 3 mouse brains of each genotype that had been postfixed in 4% PFA were mounted on the same slide and stained with Rabbit anti-GFAP (Dako #Z0334) (1:1000 in PBS containing 0.3% Triton X-100) overnight at 4˚C, washed, and stained with anti-Rabbit Alexa Fluor® 568 (1:500) for 1 hour at room temperature, washed, and mounted in FluorSaveTM (#345789, Millipore). Sections were imaged using a 20x objective on a Leica SP6 Confocal microscope and rendered into a maximum intensity projection. Images were converted into 8-bit images in ImageJ, filtered with the same thresholds (to 224/255 setting) and inverted prior to Sholl analysis, with the number of main branches set to 6. Data output from SNT Sholl Analysis software was analyzed in Python (version 3.8.12; https://www.python.org/). For each sample, the cumulative sum of Sholl complexity was calculated across the full distribution of Sholl radii that were assessed (1–63 µm). Data were plotted as average ± 99% confidence intervals.

### Statistical analysis

Unless otherwise stated, all statistical analysis was performed in Python (v. 3.8.12), using the SciPy library (v. 1.10.1; https://scipy.org/). For secretome analysis, statistical significance of differential abundance between the two genotypes was determined by paired *t*-tests with Benjamini-Hochberg *post hoc* correction, with an adjusted *P*-value threshold of 0.1. The statistical significance of the relative abundance of proteins belonging to Modules 1, 2, and 7 were determined by paired *t*-test with *post hoc* Bonferroni correction. For cell area of cultured astrocytes, statistical significance between the two genotypes was tested on the cumulative probability distribution using a two-sample Kolmogorov-Smirnov test. For *in vivo* analysis of cell morphology and branching complexity, the *P*-values for measurements of maximum extension and maximum cumulative intersection numbers were calculated according to Welch’s *t*-test, while statistical differences between the two genotypes in the Scholl analysis were assessed using a two-sample Kolmogorov-Smirnov test.

## Results

### P301S astrocytes secrete a different protein profile compared to control astrocytes

Astrocytes were cultured to confluence as described previously (Sidoryk-Wegrzynowicz et al., 2017) except that microglial proliferation was prevented by supplementing the medium with PLX3397. To produce ACM for proteomic analysis, cultures were rendered serum-free and cultured in growth medium lacking serum for 4 days. ACM was spun to remove debris, concentrated about 100-fold, denatured, and run about 1 cm into a polyacrylamide gel from which Coomassie-stained proteins (**Additional Figure 1A**) were extracted for LC-MS/MS (Scheme of the process shown in **Figure 1A**).

Of the 465 mouse proteins detected by LC-MS/MS, we selected 397 proteins for further analysis, having filtered for proteins that had > 2 unique peptides and that were detected in at least 2 out of 3 independent ACM samples from P301S and C57 mice. Of the 397 proteins, 68 (17%) were significantly enriched in P301S-ACM and 26 (6.5%) were significantly enriched in C57-ACM (**Figure 1B**). Notably, P301S-ACM was deficient for Thbs1, which was present among the significantly enriched proteins in the C57-ACM, in accordance with our previous observations (Sidoryk-Wegrzynowicz et al., 2017). Principal component analysis (PCA) of the proteins represented in **Figure 1B** shows the robust separation of the two genotypes across the PC1 axis (44% of variance), while the PC2 axis separated between the three C57-derived samples (28.7% of variance); the separation of the P301S-derived samples was captured by the PC3 axis (**Additional Figure 1B**), together accounting for 95% of all variance in the data. A volcano plot (**Figure 1C** lists the significantly enriched proteins per genotype (presented as Log2-fold change of protein abundance using a false discovery rate (FDR) of < 0.1).

**Figure 2:**
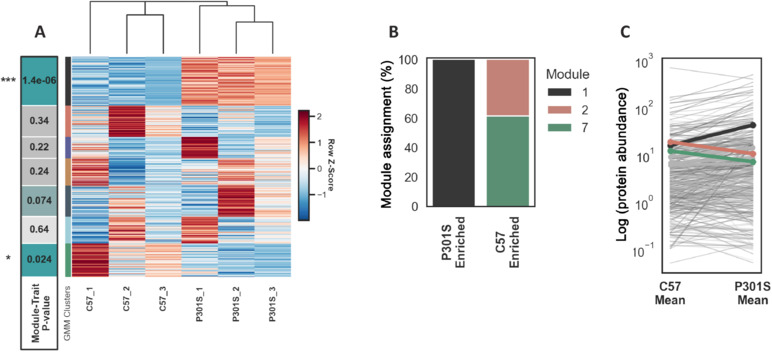
Identification of differentially expressed protein modules. (A) A heatmap depicting the Z-score-normalized protein abundance of all replicates. Correlations between columns (samples) were calculated by centroid-based Hierarchical Clustering, whilst correlations between rows (proteins) were calculated by a Gaussian Mixture Model, which identified 7 correlated modules. Only Module 1 (Grey) and Module 7 (Green) exhibited significant module-trait relationship with genotype. (B) The mean protein abundance for the protein members of the statistically significant modules. (C) Module assignments for the significantly differentially enriched proteins from Figure 1.

The robust separation of the two genotypes was corroborated by hierarchical clustering of independent replicates and depicted on a heatmap with Z-score normalized protein abundances (**Figure 2A**). Seven correlated protein modules were identified by Gaussian Mixture Model clustering (**Figure 2A**), of which two showed significant module-trait associations. Module 1 was significantly associated with the P301S genotype and contained 100% of the proteins significantly enriched in the P301S-ACM (**Figure 2B** and **C**). Conversely, Module 7 was significantly associated with the C57 genotype, and contained 60% of the proteins significantly enriched in the C57-ACM; the remaining 40% of significant C57-ACM-enriched proteins were assigned to Module 2.

**Figure 3:**
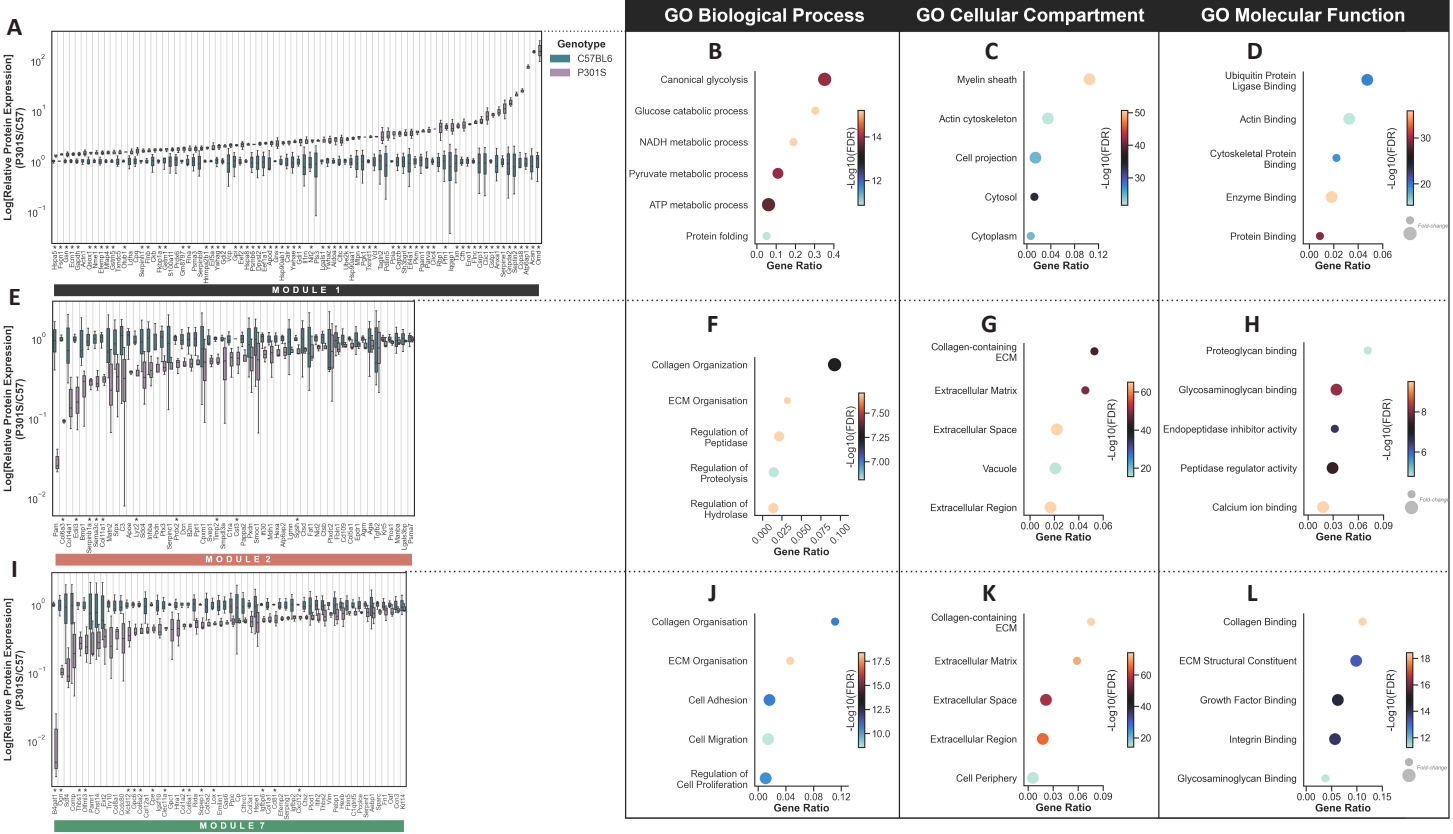
Functional annotation of protein modules. (A, E, I) Relative protein abundance for members of Module 1 (A), Module 2 (E), and Module 7 (I); *significantly differentially-enriched proteins (*P* < 0.05, paired *t*-test with *post hoc* Bonferroni correction) marked under the X axis. (B–D) Gene Ontology (GO) annotations associated with proteins in Module 1: Biological Process (B) Cellular Compartment (C) and Molecular Function (D). (F–H) Gene Ontology (GO) annotations associated with proteins in Module 2: Biological Process (F) Cellular Compartment (G) and Molecular Function (H). (J-L) Gene Ontology (GO) annotations associated with proteins in Module 7: Biological Process (J) Cellular Compartment (K) and Molecular Function (L). Circle size reflects the protein fold-change (P301S/C57) and circle color reflects the –Log10(false discovery rate) of enrichment within each GO annotation.

The constituent proteins of these three modules were submitted for functional enrichment analysis using STRING-DB. The relative abundance of proteins belonging to Module 1, Module 2, and Module 7 (**Figure 3A**, **E**, and **I**) are shown normalized to C57-ACM proteins, with significant differences marked by an asterisk below the X axis. Gene Ontology (GO) annotations associated with proteins in Module 1, Module 2, and Module 7 are shown in the adjacent panels in **Figure 3**, where the circle size reflects the protein fold-change (P301S/C57) and circle color reflects the –Log10(FDR) of enrichment for each GO annotation. We assessed the enrichment of the Biological Pathways, the Cellular Compartment and the Molecular Function GO annotations. The family of proteins enriched in P301S-ACM (Module 1) are notably cytoplasmic and are fundamental to the control of basal metabolism and energy demand (Glycolysis, Glucose catabolic process, metabolism of NADH, Pyruvate, and ATP) (see **Additional Figure 2** for a STRING diagram of the GO: ATP Metabolic Process members). By contrast, proteins enriched in C57-ACM (Module 7) belong to collagen and extracellular matrix (ECM) organization as well as cellular processes linked to ECM, such as adhesion, migration, and proliferation. The other C57-ACM enriched proteins list (Module 2) also includes collagen and ECM organization as principal processes, as well as regulatory enzymes linked to protein and catabolic metabolism (peptidases, proteolysis, hydrolysis). Catalysis of ECM components is likely necessary for the progression of some of the processes described in module 7, such as adhesion and migration (see **Additional Figure 3** for a list of ECM members).

**Figure 4:**
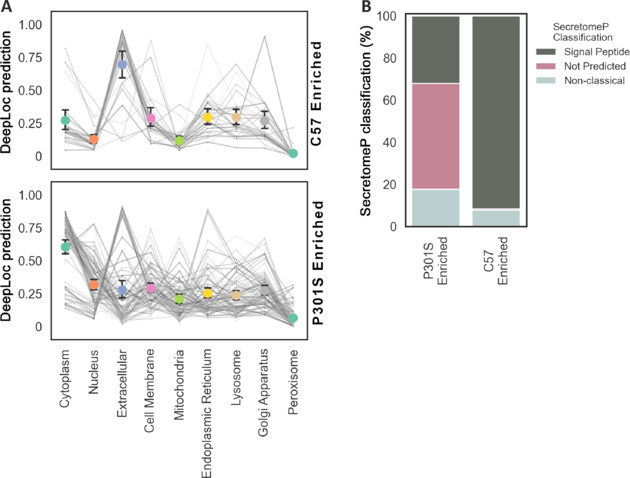
Bioinformatic prediction of protein localization and secretion. (A) DeepLoc predictions of subcellular compartment localization for the proteins significantly enriched in C57 (top) or P301S (bottom); colored points represent the mean prediction score for each subcellular compartment (± standard deviation). (B) SecretomeP classifications for the predicted mechanism of secretion based on the presence of signal peptides or putative non-classical secretion; a ‘Not Predicted’ classification means that the protein has not met these two criteria.

A striking observation that separates the proteins in the P301S-ACM from those of C57-ACM is the abundance of proteins that were associated with intracellular pathways. We submitted protein FASTA sequences to open-source bioinformatics programmes to predict protein localization and secretion, using DeepLoc (**Figure 4A**) or SecretomeP (**Figure 4B**), respectively. Confirming our observations from the GO pathway annotations, the major localization of C57-ACM-enriched proteins was predicted to be the extracellular space, whilst P301S-ACM-enriched proteins were predicted to be cytoplasmic (**Figure 4A**). SecretomeP yielded secretion predictions that were consistent with DeepLoc: over 90% of the proteins in C57-ACM were predicted to contain signal peptides, with the other 10% predicted to be released via a non-classical route. This contrasts with P301S-ACM-enriched proteins, where only 30% were predicted to contain signal peptides and 20% predicted to be secreted via a non-classical route, whilst the remaining 50% were not predicted to be secreted (**Figure 4B**). P301S astrocytes were not intrinsically damaged, as the majority of proteins (75%) were not significantly different between the two genotypes, 61% of which were predicted by UniProt to have either signal peptides or were known to be secreted, as shown in **Additional Figure 4**.

### P301S astrocytes have a less branched and more immature morphology

**Figure 5:**
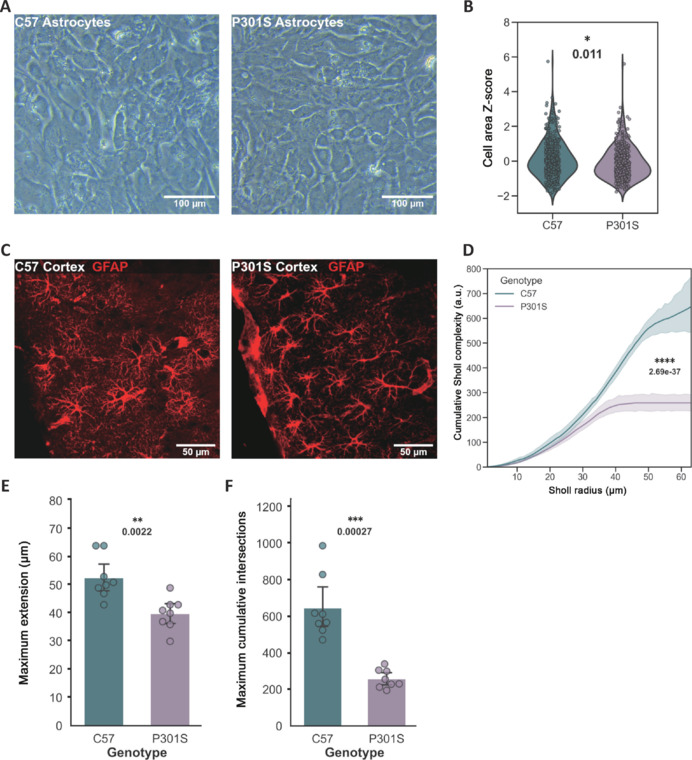
Morphological characteristics of cultured P301S and C57 astrocytes *in vitro* and *in vivo*. (A) Representative image of a random field of cultured astrocytes from C57 and P301S mice captured just before astrocyte-conditioned media collection. (B) Violin plots of areas of cultured astrocytes from two independent cultures. Areas were normalized by z score. Cumulative probabilities of all the cells counted were used to analyze significance using the Kolmogorov-Smirnoff test (*n* = 240 cells per genotype, *P* = 0.011). (C) Representative images of cortical astrocytes from 5-month-old C57 or P301S mice immunostained for glial fibrillary acidic protein (GFAP; red). (D) Cumulative number of intersections counted by Sholl analysis (7–8 cells per genotype from two different mouse brains, *P* < 0.0001, Kolmogorov-Smirnoff test). Plateau starting around 40 µm in P301S plot signifies that there were no further intersections beyond this point. (E) Maximum extension of astrocytes from origin of circle to 63 µm. *P* < 0.0022 (Welch’s *t*-test, *n* = 8 per genotype). (F) Total number of intersections in the same circle area as in E. *P* < 0.00027 (Welch’s *t*-test, *n* = 8 per genotype).

Previously we noted that P301S astrocytes are deficient in key functional proteins, such as glutamate transporters glutamate-aspartate transporter (GLAST), glutamate transporter-1 (GLT-1), and glutamine synthetase (GS). They were also more proliferative (Sidoryk-Wegrzynowicz et al., 2017). To investigate whether P301S-derived astrocytes showed other abnormalities that indicate loss of function, we measured the sizes of the astrocytes as an indication of maturity, since astrocytes develop during the first weeks from more compact morphologies with few branches to mature highly complex branched morphologies (Felix et al., 2021). Micrographs of cultured astrocytes from C57 and P301S mice captured at confluence, just before conditioned medium collection show no apparent differences in general morphology, density, purity, or viability of the two cultures (**Figure 5A**). However, measurement of cell areas showed that a significant proportion of P301S-derived astrocytes (*P* = 0.011, tested on cumulative probability distributions) were smaller than their C57-derived counterparts (**Figure 5B**), indicating a possible developmental deficit that is retained during long-term culture given that the main phase of astrocyte development occurs postnatally. Morphological differences were also apparent in less dense cultures of astrocytes prior to confluence, with P301S-derived astrocytes displaying fewer fine processes than age-matched controls (**Additional Figure 5A**). To investigate whether this also occurred *in vivo*, astrocytes in sections from the superficial cortex of 5-month-old C57 and P301S mice, when there are abundant neurofibrillary tangles and neuronal loss becomes evident (Hampton et al., 2010), were stained for GFAP (**Figure 5C**). Using Sholl analysis, we not only observed a similar decrease in the size of astrocytes in P301S mice compared to C57 mice (**Figure 5E**, maximum extension (µm): P301S 39.9 ± 5.7, C57 52.6 ± 7.6, *P* = 0.0022, mean ± standard deviation of the mean, *n* = 8 cells per genotype from 2 independent biological samples), but also less morphological complexity, with C57-derived astrocytes displaying very fine arbors with extensive branch segmentation typical of mature astrocytes compared to the P301S-derived astrocytes (**Figure 5D**, *P* = 2.7e-37, Kolmogorov-Smirnov test; **Figure 5F**, maximum cumulative intersections: P301S 258 ± 51.2, C57 646.8 ± 173.6, *P* = 0.00027; see **Additional Figure 5** for images of astrocytes prior to and following Sholl analysis). Interestingly, the density of astrocytes in the cortex of P301S mice does not appear to be altered compared to C57 mice (*n* = 2 independent biological samples of each genotype, 17–20 astrocytes per section, C57 17 ± 1.4 *vs.* P301S 19 ± 1.4, *P* = 0.292). Hence, cortical astrocytes from, and in, the P301S mouse appear to have some structural deficits.

## Discussion

Our results show that cultured astrocytes from the cortex of 1-week postnatal P301S mouse brain produce a distinct secretome signature, characterized by an enrichment of intracellular proteins that participate in the regulation of metabolic processes and energy demand, and a deficiency in proteins that are part of, and participate in, ECM organization.

The significant association of P301S-enriched proteins with intracellular functions, and the lack of predicted secretory peptide sequences, highlights a gross dysfunction in astrocyte secretion before the onset of obvious neuronal tau pathology. However, it is not clear why these proteins are being detected in the extracellular milieu. We did not see evidence of increased astrocyte death in cultures derived from P301S mice and postulated that increased cell death would also be supported by the detection of a broader range of significantly enriched cytoplasmic proteins. However, the P301S-enriched proteins observed and presented herein consist primarily of cytoplasmic metabolic proteins, specifically glycolytic enzymes. Furthermore, most of the detected proteins (75%) were common between P301S and C57 ACMs, of which 61% were designated by UniProt as being secreted. As such, P301S-ACM, although largely similar to the C57-ACM, was uniquely enriched for specific metabolic enzymes and likely to be from living cells. Because we did not separate extracellular vesicles (EVs) from our ACMs, a possible mechanism that explains the presence of cytoplasmic proteins is their increased export via EVs. Although we did not find any of the markers of exosomes amongst the 397 proteins (Ng and Ng, 2022) in keeping with a recent report that also did not detect exosomes but rather small fibulin-rich EVs, which were suggested to promote synapse formation via fibulin-2-mediated activation of transforming growth factor-beta signaling (Patel and Weaver, 2021). Thus, the presence of intracellular proteins in the P301S-enriched ACM could possibly be attributed to small EVs with altered cargo that is reflective of changes in the metabolism of astrocytes from P301S mice or a compensatory mechanism. Matafora et al. (2023) analyzed the secretome of astrocytes exposed to media from neurons that express the human mutant amyloid precursor protein (APPSwe) transgene and also found that over two-thirds of amyloid-β (Aβ)-modulated ACM were secreted via non-conventional secretory pathways. It has been proposed that glycolytic enzymes released by cells in vesicles can provide metabolic support to neighboring cells (Fridman et al., 2022; Ma et al., 2023). Since P301S-derived astrocytes are deficient in maintaining glutamate homeostasis because of reduced expression of GLT-1, GS, and GLAST (Sidoryk-Wegrzynowicz et al., 2017), perhaps secretion of metabolically-charged proteins is some form of compensation. Another possible explanation is that the cytoplasmic-derived proteins were sourced from the proliferative population of the P301S-derived astrocytes, proliferation being enhanced in the P301S-derived astrocytes (Sidoryk-Wegrzynowicz et al., 2017), a phenotype associated with neurodegeneration (Ng and Ng, 2022) and injury (Hatten et al., 1991).

The main proteins whose representation is impoverished in P301S-ACM are ECM proteins that are secreted via conventional secretion mechanisms, predicted to either have signal sequences or to be found in the ECM. Indeed, the remaining 60% of the shared secreted proteins contain such motifs, indicating that there is no deficit in conventional secretion per se. Amongst the proteins we studied previously, there was a reduction in Thbs1, which agrees with our previous observation that astrocytes from P301S mice are deficient in secreting Thbs1, thereby reducing cultured neuron synaptogenesis and survival. It is interesting that reduced Thbs1 secretion was also noted by Matafora et al. (2023) in response to Aβ. Other reduced proteins are mainly collagen subtypes that are reported to be neuroprotective against neurodegeneration (Milioto et al., 2024) and are most prominent in supporting the neurovascular unit. Indeed it has been shown that pericytes are coated with less collagen during the late Braak stages of Alzheimer’s disease patient brains in both the hippocampus and the cortex (Kirabali et al., 2020). Loss of Thbs1 secretion in astrocytes has been attributed to high glucose associated with TLR9 activation-mediated reactive oxygen species (ROS) signaling (Zhao et al., 2018). ROS signaling was also implicated in Aβ-dependent astrocyte changes (Matafora et al., 2023). Whether ROS signaling is a feature of P301S-astrocytes which could explain changes in secretion patterns remains to be explored.

Alongside these functional deficits and the altered secretome, we found that P301S-derived astrocytes are smaller and less morphologically complex not only in culture but also *in vivo*. Astrocytes increase their size and complexity during the first weeks of postnatal development in response to increasing synaptic maturation (Felix et al., 2021). Given that transgenic tau is expressed in neurons from postnatal day 1 (Scattoni et al., 2010) and neuronal loss begins at 3 months of age in the P301S-mouse cortex, particularly in the superficial layers (Hampton et al., 2010), this neuronal dysfunction may explain the reduced complexity of astrocytes *in vivo*. Interestingly, we did not find a difference in the density of astrocytes between C57 and P301S mouse brains. A deficit in Thbs1 could be one factor that influences the shape of the astrocytes without regulating their number. A reduced occupation of the territory of cortical astrocytes was also reported in the amyloid APP/PS1 mouse model (Freeman, 2010). The finding that cultured P301S-derived astrocytes retain a morphological distinction despite being separated from their natural environment may hint at deeper epigenetic changes, such as those found in AD brains and attributed to non-neuronal cells, such as astrocytes (Shireby et al., 2022). Regarding the size differences *in vitro*, it is unlikely that the astrocytes have changed their volume because of direct osmotic or ischemic stress (Risher et al., 2009) or due to an altered input from microglia, since microglia were removed using PLX3397 starting a few weeks before the time when ACM was collected. The astrocytes do not express or contain mutant tau (Sidoryk-Wegrzynowicz et al., 2017), which is expressed under a neuron-specific promoter, and so mutant tau is an unlikely cause of size change. It is possible that some of the astrocytes (those that are morphologically altered) are sensing some kind of stress that spreads to other astrocytes, similar to the case where ER-stressed astrocytes secrete a molecule(s) with lipid characteristics which regulate both inflammatory and ER stress responses in other astrocytes (Sprenkle et al., 2019). A stress signal may have also been originally imparted by the neurons harboring transgenic tau (Batenburg et al., 2023). It will be interesting to test the astrocytes for metabolic changes, and the ACM for metabolic and lipidomic abnormalities (Guttenplan et al., 2021) (and the transcriptome) as clues for the mechanisms underlying the disability of P301S-derived astrocytes to maintain a normal secretome and determine whether these are present in human brain.

In terms of biomarkers, the reduced Thbs1 secretion observed in this study could be an important predictor of reduced neuronal function and survival in general (Sidoryk-Wegrzynowicz et al., 2017), just as elevated astrocyte-derived Thbs1 is important for the recovery of excitatory synaptic inputs onto axotomized motor neurons (Tyzack et al., 2014). Could Thbs1 perturbations be detected in the cerebrospinal fluid (CSF) and could restoring Thbs1 secretion be a useful treatment? Two studies have identified separate pathology-related perturbations of CSF Thbs1 levels, demonstrating that altered cellular secretion in the brain parenchyma can reflect its concentration in the CSF (Yang et al., 2015; Chen et al., 2016). As such, reduced CSF Thbs1 could be explored as a potential biomarker of the altered astrocyte state described herein. In terms of treatment, it is of interest that presymptomatic pediatric spinal muscular atrophy patients exhibited reduced Thrombospondin4 (Thbs4) presence in the CSF, which was restored by addressing the underlying cellular dysfunction with SMN2 antisense oligonucleotides (Dobelmann et al., 2024). This is of interest because, at least in pediatric spinal muscular atrophy, Thbs4 reduction occurs in the asymptomatic phase of pathology, as we observe with Thbs1 in our mouse model of tau pathology. Restoring conventional astrocyte secretion by addressing the underlying cause of Thbs1 deficiency could yield therapeutic benefit, which is also preferable over exogenous Thbs1 application, as it may be an important contributor to the development of glioblastoma (Bikfalvi et al., 2024).

Regarding the identity of other cytoplasmic proteins in the ACM, we have not identified any specific protein that could prove to be a biomarker for this state of astrocytes; rather, it is the phenomenon of increased cytoplasmic protein secretion that defines this abnormal astrocyte state. Typically, the utility of a given protein as a CSF biomarker appears to be determined by the presence of signal peptides and non-cytoplasmic subcellular localization (Waury et al., 2023). However, our data suggest that the importance of unconventional protein secretion should not be overlooked. Interestingly, GFAP was not detected in the P301S-derived astrocyte secretome although its expression is elevated in the cortex of adult P301S mice (Hampton et al., 2010; Sidoryk-Wegrzynowicz et al., 2017). While elevated plasma GFAP has been considered to be a biomarker of astrogliosis in several human neurodegenerative diseases, the predictive value of serum GFAP remains unclear as there is no strict correlation between serum GFAP and disease penetrance or prognosis (Kumar et al., 2023).

This study has some limitations: we did not perform a biochemical confirmation of proteins found to be differentially secreted and their impact on astrocyte and neuronal/brain function. We did not extend our study to other models of tauopathy or investigate whether astrocytes in the human brain behave similarly. It will also be important to understand the mechanisms underlying the unconventional secretion we observed to gain better insight into disease mechanisms.

In summary, our data support the alleviation of the type of astrocyte dysfunction we describe herein as a potentially important target when developing treatments for tauopathies.

## Additional files:

***Additional Figure 1:*** *Images of gels use for protein extraction and three-dimensional representation of PCA results.*

Additional Figure 1 Images of gels use for protein extraction and three-dimensional representation of PCA results.
(A) Images of gels stained with Coomassie blue prior to extraction of proteins for liquid chromatography-mass spectrometry. (B) Three-dimentional principal component analysis plot showing the distribution of P301S samples long the 3rd dimension. Altogether, this analysis accounts for 95% of all astrocyte-conditioned media proteins.

***Additional Figure 2:*** *STRING diagram of the secreted proteins enriched in the P301S-astrocyte-conditioned media that are members of the GO: ATP Metabolic Process.*

Additional Figure 2 STRING diagram of the secreted proteins enriched in the P301S-astrocyte-conditioned media that are members of the GO: ATP Metabolic Process.
Lines are an aggregate representation of the interaction confidence (bolder lines = more confident).

***Additional Figure 3:*** *STRING diagram of the secreted proteins enriched in the C57-astrocyte-conditioned media that are members of the GO: Extracellular Matrix Organization.*

Additional Figure 3 STRING diagram of the secreted proteins enriched in the C57-astrocyte-conditioned media that are members of the GO: Extracellular Matrix Organization.
Lines are an aggregate representation of the interaction confidence (bolder lines = more confident).

***Additional Figure 4:*** *STRING diagram of the secreted proteins that are predicted using Uniprot Keyword to be Secreted (in red, 172) and/or carry a Signal peptide (in blue. 114) that are common in both C57- and P301S-astrocyte-conditioned media.*

Additional Figure 4 STRING diagram of the secreted proteins that are predicted using Uniprot Keyword to be Secreted (in red, 172) and/or carry a Signal peptide (in blue, 114) that are common in both C57- and P301S-astrocyte-conditioned media.
Lines are an aggregate representation of the interaction confidence (bolder lines = more confident).

***Additional Figure 5:*** *Images of cultured astrocytes and output of Scholl analysis.*

Additional Figure 5 Images of cultured astrocytes and output of Scholl analysis.
(A) Representative images of sparsely plated cultured cortical astrocytes 3 days after passage of confluent astrocyte cultures from 7-day-old pups, immunostained for glial fibrillary acidic protein (GFAP). C57 astrocytes show more complex morphologies, some extending fine processes (white arrows) compared to astrocytes from P301S mice. (B, C) Top panels: Representative rendered confocal images of GFAP-labelled astrocytes in the superficial cortex of (B) 5-month-old C57 mice and (C) 5-month-old P301S mice after applying equal thresholds in ImageJ prior to Sholl analysis. Below: Intersection analysis showing Scholl overlay output of the cells circled in B and C.

## Data Availability

*All relevant data are within the paper and its Additional files*.
