# Supplementary figures and images for: Astrocytes from P301S Tau mice exhibit non-canonical protein secretion and reduced morphological complexity
Source: Neural Regen Res. 2025 Aug 13;21(7):3149–55. doi: 10.4103/NRR.NRR-D-24-01598 (PMC13378943; doi:10.4103/NRR.NRR-D-24-01598)

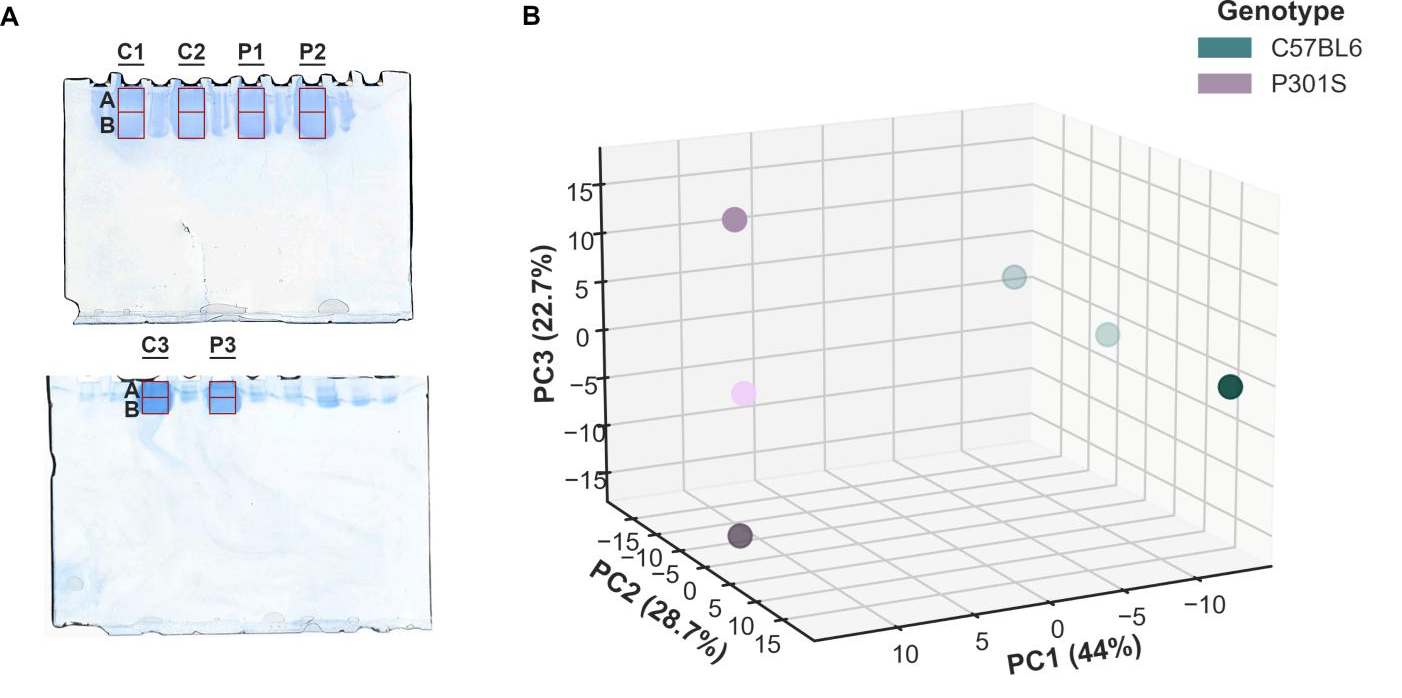

Supplement: Supplementary file 1 [file NRR-21-3149_Suppl1.tif]

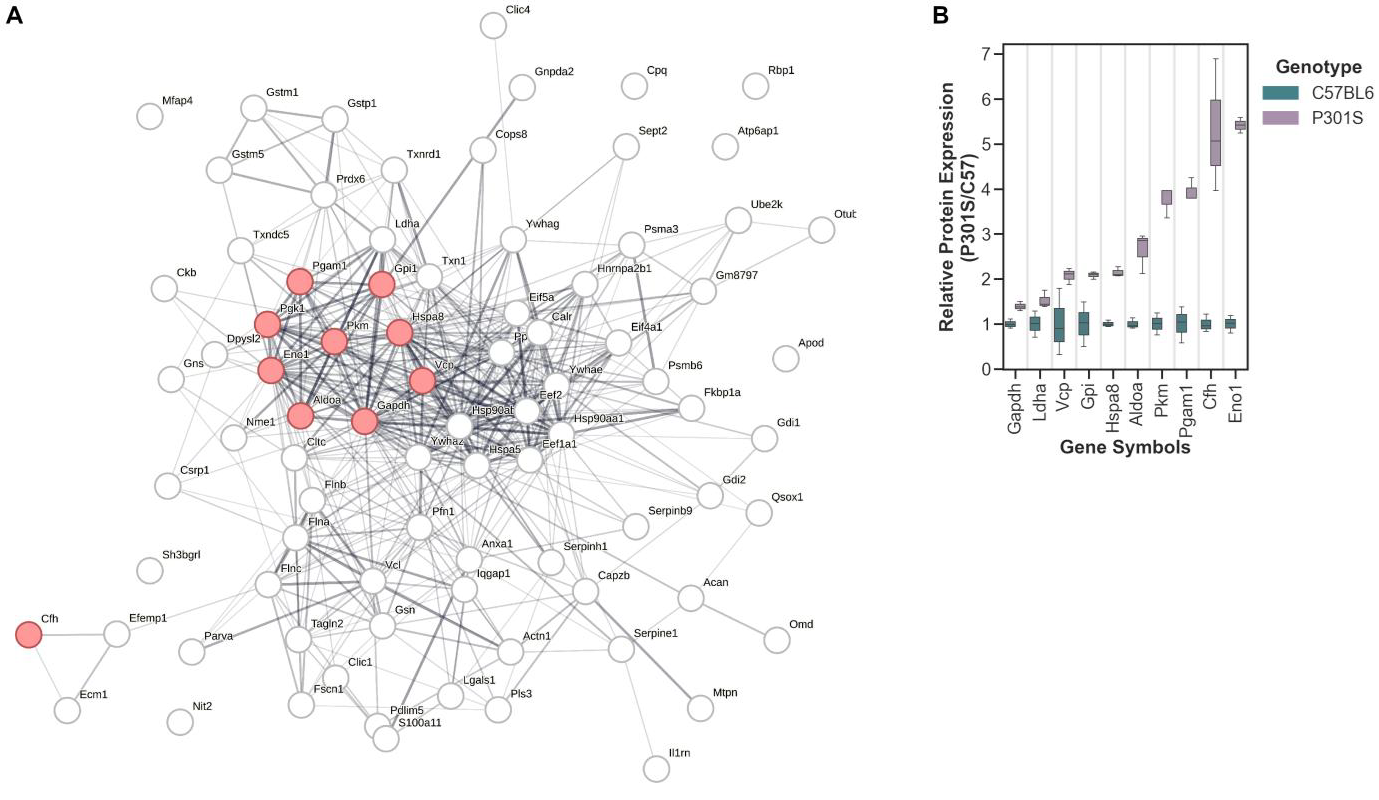

Supplement: Supplementary file 2 [file NRR-21-3149_Suppl2.tif]

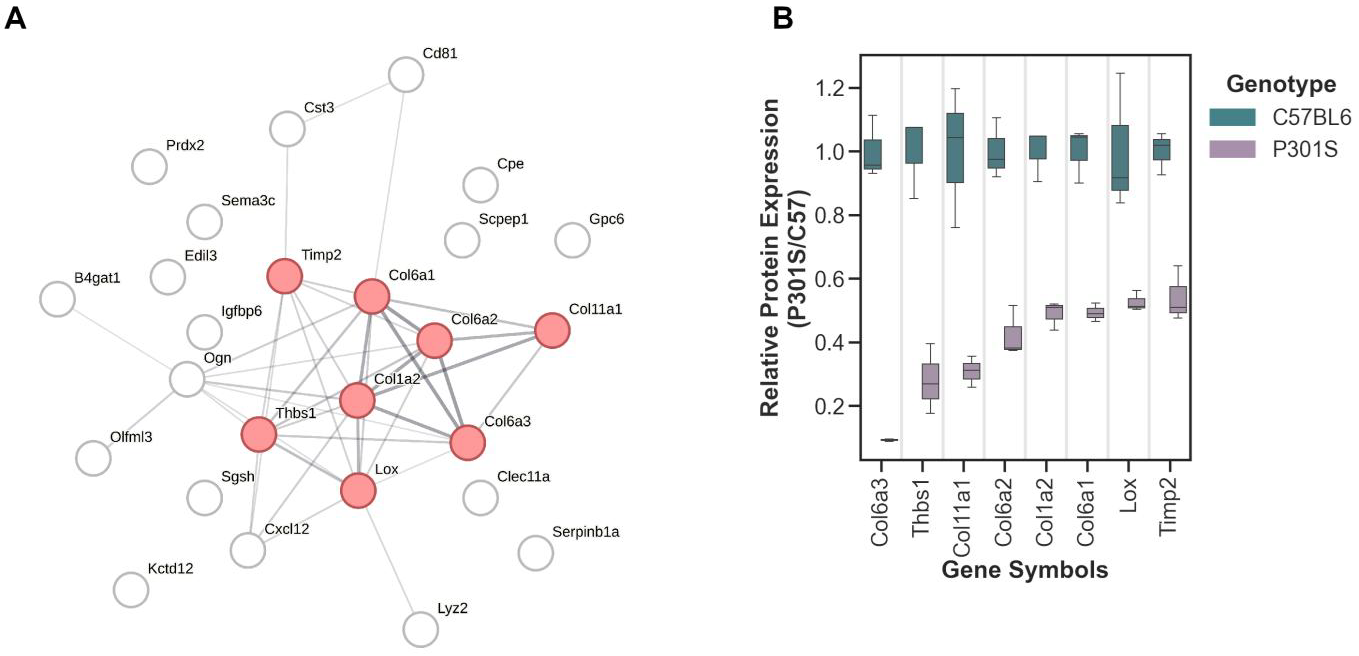

Supplement: Supplementary file 3 [file NRR-21-3149_Suppl3.tif]

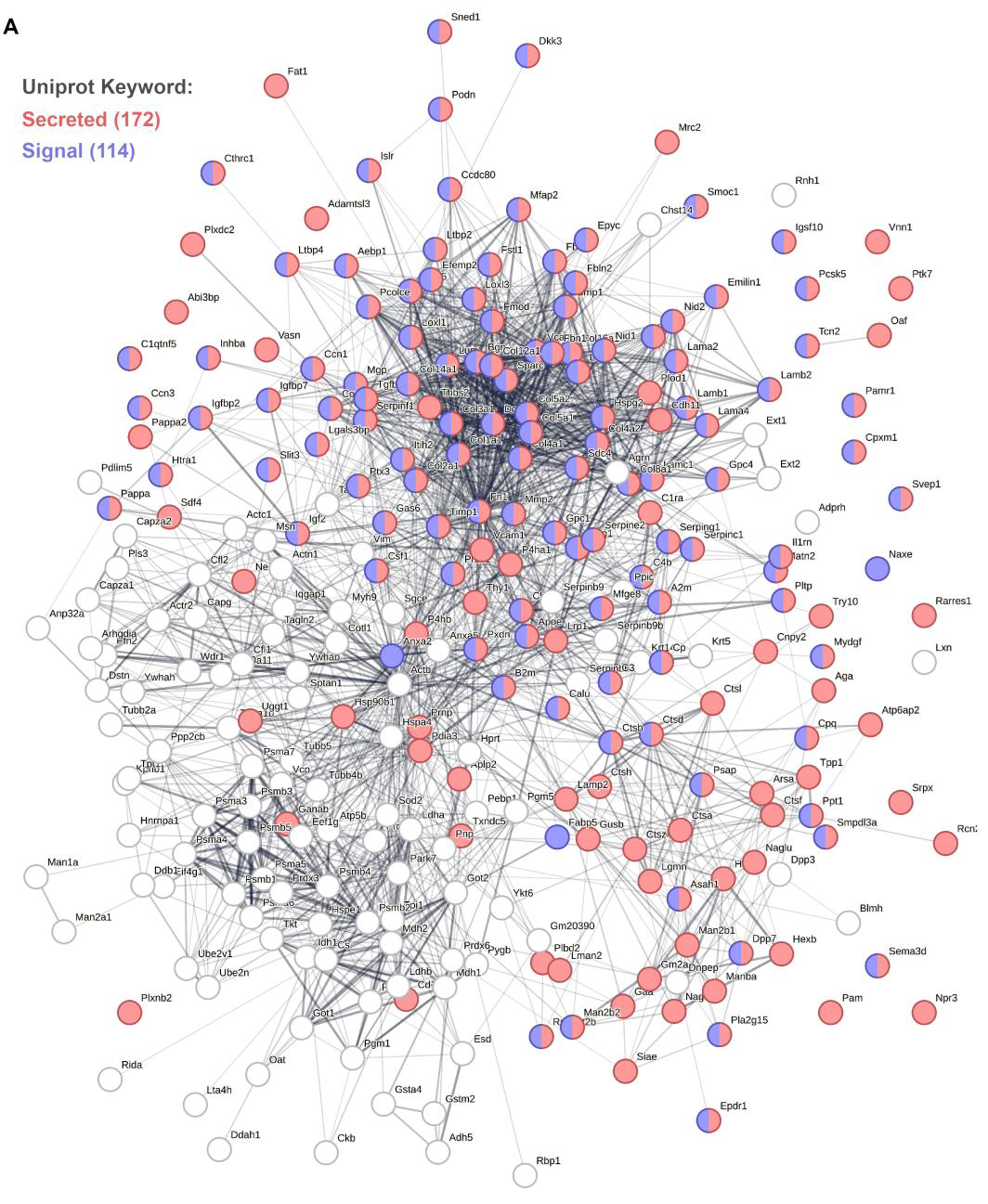

Supplement: Supplementary file 4 [file NRR-21-3149_Suppl4.tif]

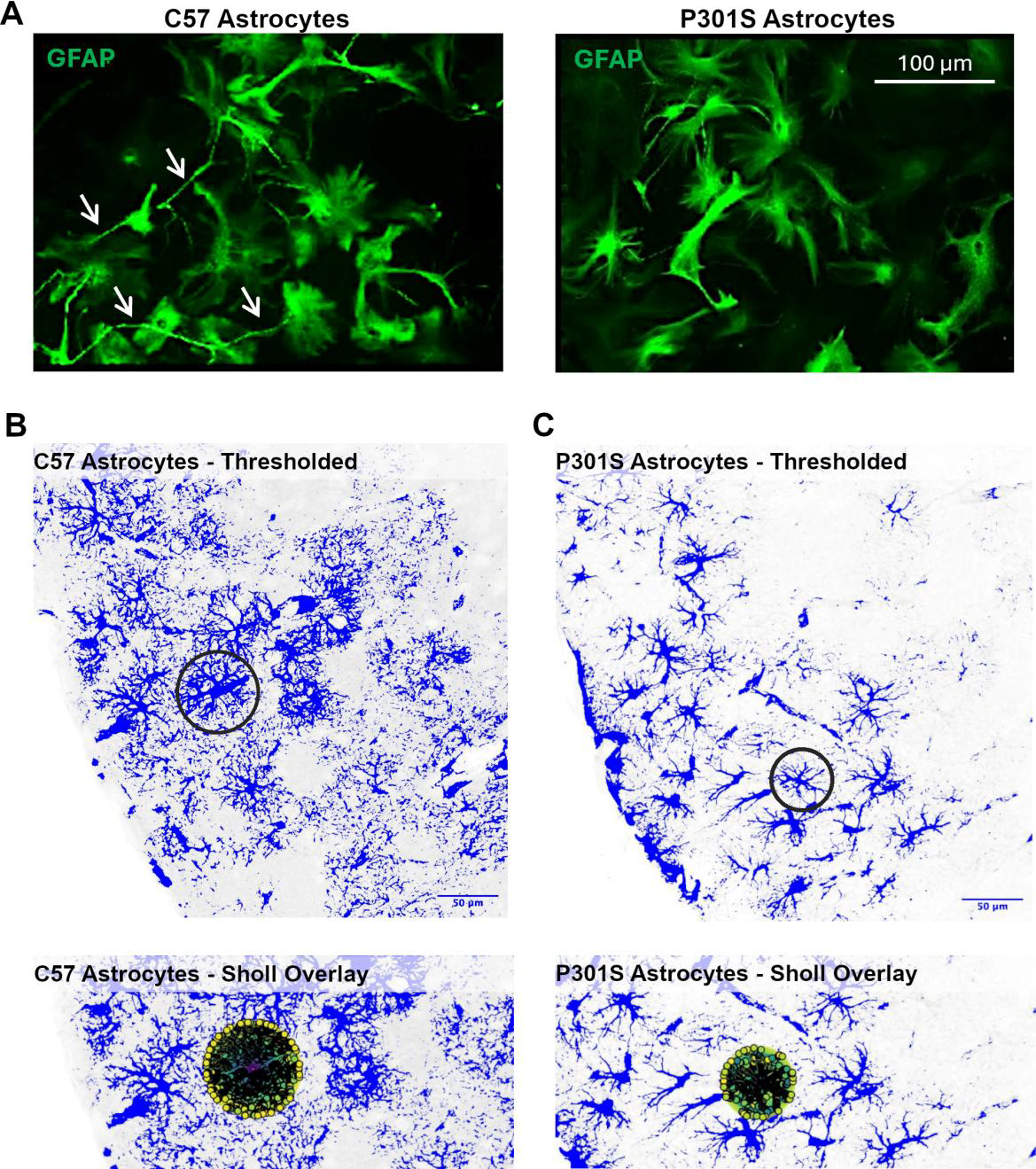

Supplement: Supplementary file 5 [file NRR-21-3149_Suppl5.tif]
